# Enhanced expression of two discrete isoforms of matrix metalloproteinase-2 in experimental and human diabetic nephropathy

**DOI:** 10.1371/journal.pone.0171625

**Published:** 2017-02-08

**Authors:** Sang Soo Kim, Nari Shin, Sun Sik Bae, Min Young Lee, Harin Rhee, Il Young Kim, Eun Young Seong, Dong Won Lee, Soo Bong Lee, Ihm Soo Kwak, David H. Lovett, Sang Heon Song

**Affiliations:** 1 Biomedical Research Institute and Department of Internal Medicine, Pusan National University Hospital, Busan, Republic of Korea; 2 Department of Pathology, Pusan National University Yangsan Hospital, Yangsan, Gyeongnam, Republic of Korea; 3 MRC for Ischemic Tissue Regeneration, Medical Research Institute, and Department of Phamacology, Pusan National University School of Medicine, Yangsan, Republic of Korea; 4 Research Institute for Convergence of Biomedical Science and Technology and Department of Internal Medicine, Pusan National University Yangsan Hospital, Yangsan, Gyeongnam, Republic of Korea; 5 The Department of Medicine, San Francisco Department of Veterans Affairs Medical Center/University of California San Francisco, San Francisco, California, United States of America; University of Louisville, UNITED STATES

## Abstract

**Background:**

We recently reported on the enhanced expression of two isoforms of matrix metalloproteinase-2 (MMP-2) in human renal transplantation delayed graft function. These consist of the conventional secreted, full length MMP-2 isoform (FL-MMP-2) and a novel intracellular **N**-**T**erminal **T**runcated isoform (NTT-MMP-2) generated by oxidative stress-mediated activation of an alternate promoter in the MMP-2 first intron. Here we evaluated the effect of hyperglycemia and diabetes mellitus on the in vitro and in vivo expression of the two MMP-2 isoforms.

**Methods:**

We quantified the abundance of the FL-MMP-2 and NTT-MMP-2 transcripts by qPCR in HK2 cells cultured in high glucose or 4-hydroxy-2-hexenal (HHE) and tested the effects of the NF-κB inhibitor pyrrolidine dithiocarbamate (PDTC). The streptozotocin (STZ) murine model of Type I diabetes mellitus and renal biopsies of human diabetic nephropathy were used in this study.

**Results:**

Both isoforms of MMP-2 in HK2 cells were upregulated by culture in high glucose or with HHE. PDTC treatment did not suppress high glucose-mediated FL-MMP-2 expression but potently inhibited NTT-MMP-2 expression. With STZ-treated mice, renal cortical expression of both isoforms was increased (FL-MMP-2, 1.8-fold; NTT-MMP-2, greater than 7-fold). Isoform-specific immunohistochemical staining revealed low, but detectable levels of the FL-MMP-2 isoform in controls, while NTT-MMP-2 was not detected. While there was a modest increase in tubular epithelial cell staining for FL-MMP-2 in STZ-treated mice, NTT-MMP-2 was intensely expressed in a basolateral pattern. FL-MMP-2 and NTT-MMP-2 isoform expression as quantified by qPCR were both significantly elevated in renal biopsies of human diabetic nephropathy (12-fold and 3-fold, respectively).

**Conclusions:**

The expression of both isoforms of MMP-2 was enhanced in an experimental model of diabetic nephropathy and in human diabetic nephropathy. Selective MMP-2 isoform inhibition could offer a novel approach for the treatment of diabetic renal disease.

## Introduction

Diabetes mellitus (DM) has an increasing prevalence worldwide and is associated with major morbidity and mortality. According to the 2014 National Diabetes Statistics Report, over 9% of the US population (or 29.1 million people) have diabetes mellitus [1]. Diabetic nephropathy is an increasingly frequent microvascular complication of diabetes mellitus and the leading cause of end-stage renal disease (ESRD) worldwide, including Korea. Multiple pathologic processes contribute to the development and progression of diabetic nephropathy, including acute and chronic hyperglycemia, hypertension, dyslipidemia, oxidative stress, inflammation and genetics [2]. At present, it is not possible to predict in which patients diabetic nephropathy will develop, presumably due to the complexity of the individual contributing factors.

Matrix metalloproteinase-2 plays a major role in the pathogenesis of cardiovascular and renal disease [3–7]. In terms of the kidney, enhanced expression of MMP-2 has been described in several experimental models of renal injury, including ischemia/reperfusion injury [8–10]. In addition, transgenic renal proximal tubule-specific expression of the secreted, full length MMP-2 (FL-MMP-2) isoform is sufficient to induce all of the common features of progressive human renal disease, including glomerulosclerosis, tubular atrophy, interstitial fibrosis and inflammation [3]. These observations suggest that MMP-2 may represent a common pathway for the development of progressive renal disease.

We recently reported on the identification and characterization of a novel intracellular isoform of MMP-2 generated by oxidative stress-mediated activation of an alternate promoter located in the first intron of the MMP-2 gene [11]. This event leads to the synthesis of an **N**-**T**erminal **T**runcated MMP-2 isoform (NTT-MMP-2) that localizes to mitochondria and triggers a primary innate immune response resulting in inflammation and cell death [11). Both the acute and chronic hyperglycemia of diabetes mellitus is associated with increased oxidative stress [12, 13]. We hypothesized, in the setting of experimental and clinical diabetic nephropathy, that hyperglycemia-mediated oxidative stress enhances tubular epithelial expression of both MMP-2 isoforms, leading to cellular injury. The experiments outlined in this report confirm our hypothesis as detailed below.

## Materials and methods

### HK2 cell culture experiments

The immortalized human proximal tubule cell line HK2 was obtained from ATCC (Manassas, VA, USA) and cultured in Dulbecco’s modified Eagle’s medium (DMEM) containing 5.5 mM D-glucose, 10% heat-inactivated fetal bovine serum and antibiotics-antimycotics (Gibco^®^/Life Technologies, Paisley, UK). Cells were incubated at 37°C in a humidified incubator with 5% CO_2_. For experimental studies, HK2 cells were cultured with different concentrations of D-glucose (5mM, 30mM) for 2, 24 and 48h, respectively. Additionally, 4-hydroxy-2-hexenal (HHE) was obtained from Cayman Chemical, Inc. (Ann Arbor, MI, USA). HHE induces lipid peroxidation and is a stimulant of oxidative stress. HK2 cells were cultured with 10μM HHE for 2, 24 and 48h and compared with a non-treated control group. Control cells were harvest at 2hr because the expression of MMP-2 was not significantly different according to varying time point (data not shown). RNA was extracted from cells using TRIzol^®^ reagent (Invitrogen; Life Technologies Korea, Seoul, Korea) according to the manufacturer’s protocol and processed to measure the expression of two MMP-2 isoforms using a quantitative real-time polymerase chain reaction (qPCR) detailed below. An antibody to p65-NFkB (ser536) was obtained from Cell Signaling Technology (Danvers, MA, USA). The antioxidant and NF-kB inhibitor pyrrolidine dithiocarbamate (PDTC) was obtained from Sigma-Aldrich, Inc. (St. Louis, Mo, USA). PDTC treatment was performed at 50 μM 1h prior to D-glucose stimulation.

### Immunofluorescence studies of HK2 cells

HK2 cells were grown on sterilized coverslips in 6-well plates, and then washed with PBS, fixed with 4% buffered paraformaldehyde (4°C) for 20 min and permeabilized in 0.1% Triton X-100/PBS 1X for 10 min. Subsequently, the cells were incubated with a FL-MMP-2-specific monoclonal antibody (prediluted) directed against the N-terminal domain (ab50441, Abcam, Cambridge, UK). A NTT-MMP2-specific antibody targeting the S1’ substrate binding loop was used at 20 μg/ml 0.1% BSA/PBS [11, 14]. Both primary antibody incubations were performed at 4° C overnight [11]. The coverslips were then incubated with the FITC-conjugated secondary antibody (Molecular Probes) for MMP-2 (1:500, Alexa fluor® 488 goat anti-mouse IgG); for NTT-MMP-2 (1:800, Alexa fluor® 488 rabbit anti-goat IgG) at room temperature for 30 min. Cell nuclei were stained with DAPI (Molecular Probes, Oregon, USA) 1:2000 for 10 min. The coverslips were mounted onto microscopy glass slides with mounting solution. Images were obtained with a Leica TCS-SP8 confocal microscope (Leica, Mannheim, Germany). Immunofluorescence intensity was measured using the Image J program (National Institutes of Health, Bethesda, MD, USA).

### Murine model of diabetic nephropathy

The animal protocol (2014–069) used in this study was reviewed and approved by Pusan National University–Institutional Animal Care and Use Committee (PNU-IACUC) on their ethical procedures and scientific care. Mice were randomized into control and diabetic groups. Diabetes mellitus was induced by five daily intraperitoneal injections of streptozotocin (40mg/kg in citrate buffer, pH4.5, Sigma-Aldrich) in 8-week-old C57/BL6 male mice. Control mice received citrate buffer alone. After 1 week, blood glucose levels were measured using a blood glucometer by tail vein puncture blood sampling. Body weight was measured weekly and blood glucose concentrations were monitored every four weeks to confirm hyperglycemia. Mice were sacrificed under isoflurane anesthesia at 12 weeks and 24 weeks, respectively. Each group was comprised of eight mice. Kidneys were perfused with 4° C phosphate-buffered saline (PBS) and then excised. The renal cortex was harvested after excision of the medullary portion. Half of the kidney was fixed in 10% neutralized formalin for immunohistochemical study. The remaining portions were used for Western blot, zymography and qPCR analysis as detailed below.

### Quantitative real-time Reverse Transcriptase-Polymerase Chain Reaction (PCR) analysis

The measurements of FL-MMP-2 and NTT-MMP-2 mRNA expression were conducted with quantitative RT-PCR. Total RNA was isolated using TRIzol^®^ reagent (Invitrogen; Life Technologies Korea, Seoul, Korea) according to the manufacturer’s protocol. In brief, 2 μg of total RNA was used to synthesize cDNA using oligo-dT primers and M-MLV RTase (Promega, Madison WI, USA). The reaction was performed at 42℃ for 1 h. The primers used for real-time PCR are summarized in Table 1. The amplification reaction was conducted with 40 cycles (95°C for 15 sec; 60°C for 45 sec; and 72°C for 1 min) using Fast Start Universal SYBR Green Master Mix (Rox dye, Roche) on an ABI 7500 Real-time PCR System (Applied Biosystems). For a housekeeping internal control, β-actin or ribosomal 36B4 mRNA were quantified in parallel with the target genes and all products were verified using melting curve analysis (95°C 15sec, 60°C 15sec, 95°C 15sec). Normalization and fold-change for each of the genes were calculated using 2^-ΔΔCT^ method.

**Table 1:** Quantitative polymerase chain reaction primer sequences.

Genes	Forward (5’→3’)	Reverse (5’→3’)
Full length MMP-2 (human)	TCGCCCATCATCAAGTTCCC	GGGCAGCCATAGAAGGTGTT
N-terminal truncated MMP-2 (human)	GCTGTATGTCCTGTCGCT CAA CT	GGGCAGCCATAG AAGGTGTT
Full length MMP-2 (mouse)	GACCTCTGCGGGTTCTCTGC	TTGCAACTCTCCTTGGGGCAGC
N-terminal truncated MMP-2 (mouse)	GTGAATCACCCCACTGGTGGGTG	TTGCAACTCTCCTTGGGGCAGC
36B4 (human control)	GCGACCTGGAAGTCCAACTAC	ATCTGCTGCATCTGCTTGG
β-actin (mouse control)	CTCTCTTCCAGCCTTCCTTCC	CTCCTTCTGCATCCTGTCAGC

### Western blot methods

Proteins were extracted from the excised renal cortex with a protein extraction solution (PRO-PREP™; iNtRON Biotechnology, Korea). The protein concentration was measured by the Bradford method (Bio-Rad Protein Assay, Bio-Rad Laboratories Inc., Hercules, CA, USA). Proteins were separated by electrophoresis on a 12% SDS-polyacrylamide gel and transferred onto a nitrocellulose membrane (Hybond ECL, Amersham Pharmacia Biotech Inc., Piscataway, NJ, USA). Blots were blocked for 2 h at room temperature with 5% (w/v) non-fat dried milk in Tris-buffered saline (10 mM Tris/HCl, pH 8.0, and 150 mM NaCl) solution containing 0.05% Tween-20. The membranes were immunoblotted with the following specific primary antibodies (1∶1000 dilution): rabbit polyclonal antibodies for p-NF-κB p65 (Cell Signaling Technology, Inc.), and GAPDH (Santa Cruz Biotechnology Inc., Santa Cruz, CA, USA). The blots were then incubated with the corresponding conjugated anti-rabbit immunoglobulin G-horseradish peroxidase (1∶2,000 dilution, Abcam, Cambridge. UK). Immunoreactive proteins were detected with the ECL Western blotting detection system (AB Frontier, Seoul, Korea).

### Gelatin zymography

Zymography was performed to identify MMP-2 activity in kidney tissue. Kidney cortices were extracted using lysis buffer (25mM Tris-HCl, pH 7.5, 100mM NaCl, 1% NP-40). Gelatinase activity was determined by electrophoresis on 8% SDS-PAGE) gels containing 0.1% (w/v) gelatin. After electrophoresis, the gels were washed in 2.5% Triton X-100 and incubated for 36 hours in zymography developing buffer (Komabiotech, Korea) at 37°C. The MMP-2 gelatinolytic activity was detected as clear bands on a blue background after staining the gels with Coomassie Brilliant Blue R250.

### Immunohistochemical analysis

Tissues were fixed in 10% formalin immediately after collection. Thereafter, the tissues were paraffin-processed and embedded. Immunohistochemistry was performed on formalin-fixed 3 ㎛ thick paraffin embedded sections. Sections were deparaffinized, followed by rehydration with graded ethanol to water. For immunostaining of FL-MMP-2, the sections were incubated for 30 minutes with a prediluted monoclonal anti-mouse antibody against the N-terminal sequence of the FL-MMP-2 protein (ab50441, Abcam, Cambridge, UK), followed by a 30 minute incubation with biotinylated anti-mouse IgG (Vector). For immunostaining of the NTT-MMP-2 isoform, the sections were incubated overnight at 4°C with 5 μg/ml of the isoform-specific antibody detailed above. After each primary antibody treatment, the sections were incubated for 30 minutes with secondary biotinylated antibody (Vector), followed by an incubation with Vectastain ABC complex (Vector). Imunohistochemical development was performed using VIP peroxidase substrate (Vector). All slides were counterstained with methyl green. Semi-quantitative staining grade was measured. In brief, immunohistochemical staining grade was depicted as follows: grade 0, negative staining; grade 1, weak patchy staining; grade 2, weak diffuse or dense patchy staining; grade 3, dense diffuse staining.

### Analysis of human diabetic kidney disease

This human study was approved by the institutional review board of the Pusan National University Hospital (PNUH IRB No: 2014–6) and the need for informed consent from the participants was waived as patients’ records and information were anonymized and de-identified prior to analysis. All clinical investigation have been conducted according to the principles expressed in the Declaration of Helsinki. The bio-specimens and data used for this study were provided by the Biobank of Pusan National University Hospital, a member of the Korean Biobank Network. In brief, we analyzed the expression of FL-MMP-2 and NTT-MMP-2 in archival renal biopsy specimens maintained by Pusan National University Hospital (PNUH) Department of Pathology. The study groups consisted of controls (N = 10) and diabetic kidney disease group (N = 25). Control samples were obtained mainly from patients with isolated hematuria, who had normal histopathologic findings. The experimental group (N = 25) consisted of biopsies from patients with Type 2 diabetes mellitus having evidence for diabetic nephropathy such as proteinuria, hematuria or declining renal function (Table 2). For semi-quantitative scoring of proximal tubular injury, tubular dilatation, and tubular atrophy, a 4 tier scoring was done; no change as score 0; ≤10% of renal tubules as score 1; >10% and ≤30% of renal tubules as score 2; >30% of renal tubules as score 3. Immunohistochemical staining for the MMP-2 isoforms was performed as detailed above. Quantitative expression of FL-MMP-2 and NTT-MMP-2 transcripts was assessed by quantitative RT-PCR following template extraction from surplus frozen biopsy cores stored at -80 C, using the qPCR method detailed above.

**Table 2:** The characteristics of human diabetics and control group.

Variable	DM (n = 25)	Control (n = 10)	P value
Age	45.95±13.27	29.82±12.97	0.003
Male sex	14 (66.7%)	7 (63.6%)	0.864
Serum Cr	2.33±2.08	0.74±0.16	0.002
eGFR	52.9±34.63	115.73±9.47	<0.001
DR change	13 (61.9%)	0 (0.0%)	0.001
DM duration (years)	8.71±6.9	0	<0.001

### Statistical analysis

All statistical analyses were performed using GraphPad Prism 6.0 (GraphPad Software). The Mann Whitney U test or Kruskal-Wallis test with Dunn’s multiple comparison test were used to compare among experimental groups as appropriate. Significance was defined as a *p*-value of less than 0.05. Results were presented as mean ± standard deviation for all experiments.

## Results

### High glucose and oxidative stress induce both MMP-2 isoforms in HK2 proximal tubular epithelial cells

**Fig 1:**
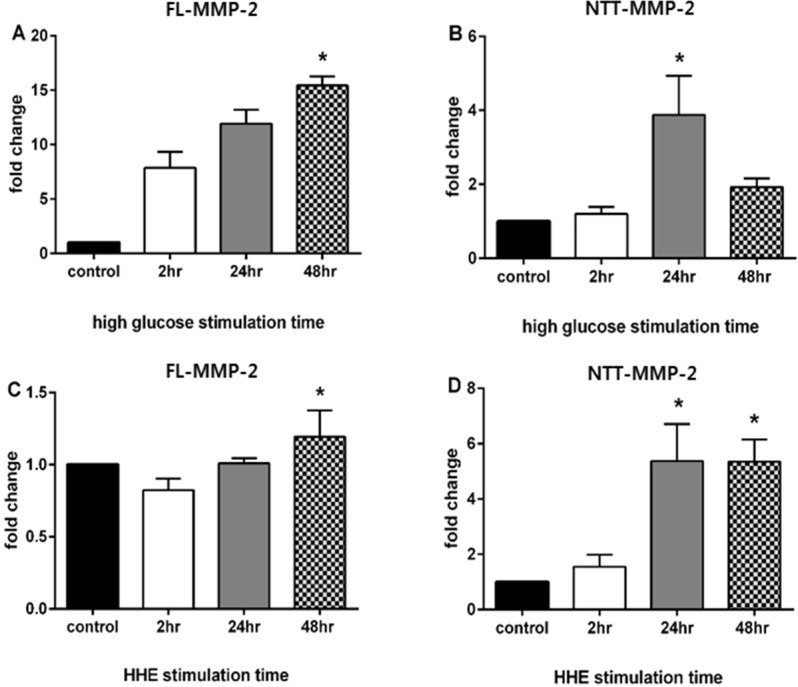
Induction of MMP-2 isoform expression in cultured HK2 cells by high glucose and HHE oxidative stress. HK2 cells were cultured in either normal glucose concentrations (5 mM) or high glucose concentrations (30 mM) and transcript levels of the FL-MMP-2 and NTT-MMP-2 isoforms determined by qPCR at 2, 24 and 48 hours of incubation. To determine the effect of oxidative stress on MMP-2 isoform expression, HK2 cells were cultured in the presence or absence of HHE (10 μM) for 2, 24 and 48 hours. All control cells were harvest at 2hr because the expression of MMP-2 was not significantly different according to varying time point (data not shown). N = 3 for all study groups. (* p<0.05)

In the case of the FL-MMP-2 isoform, gene expression increased over time and peak expression was approximately 15-fold greater than controls at 48 h (Fig 1, panel A; 15.4±0.7 fold *p*<0.001). In the case of the NTT-MMP-2 isoform, gene expression peaked at 24 h in high glucose medium and was approximately four times greater than controls (Fig 1, panel B; 3.9±0.2 fold *p*<0.001). With HHE-induced oxidative stress both FL-MMP-2 and NTT-MMP-2 transcripts were significantly elevated in HK2 cells. Specifically, FL-MMP-2 gene expression slightly increased at 48h (1.2±0.2 fold) compared with controls (Fig 1, panel C). In contrast, NTT-MMP-2 gene expression was increased by over 5-fold at 24 h and 48 h (Fig 1, panel D; 5.4±1.1, *p* = 0.001; 5.4±0.7, *p* = 0.002, respectively).

**Fig 2:**
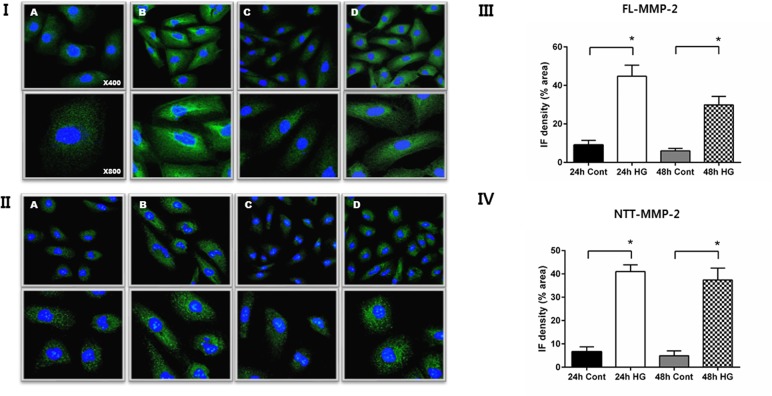
Immunofluorescent (IF) detection of MMP-2 isoform expression in HK2 cells: effect of culture in normal (5 mM) vs. high (30 mM) glucose. Panel I. IF staining for FL-MMP-2 isoform of HK2 cells cultured in normal (5 mM) glucose for 24 and 48 hours (A, C). IF staining for FL-MMP-2 isoform of HK2 cells cultured in high (30 mM) glucose for 24 and 48 hours (B, D). Upper panel x 400; lower panel x 800. Panel II. IF staining for NTT-MMP-2 isoform of HK2 cells cultured in normal (5 mM) glucose for 24 and 48 hours (A, C). IF staining for NTT-MMP-2 isoform cultured in high (30 mM) glucose for 24 and 48 hours (B, D). Upper panel x 400; lower panel x 800. Panels III and IV: Quantitation of IF staining for the FL-MMP-2 isoform (panel III) and NTT-MMP-2 (panel IV) of HK2 cells cultured in normal (5 mM) and high (30 mM) glucose medium for 24 and 48 hours. (N = 5 for all study groups; *p<0.05).

Confocal immunofluorescent microscopic findings confirmed that both isoforms of MMP-2 are induced by high glucose stimulation of HK2 cells (Fig 2, Panels I, II). However, the cellular patterns of MMP-2 protein isoform expression differed. Specifically, FL-MMP-2 protein was diffusely distributed in the cytoplasm, along with concentrated perinuclear staining. In contrast, NTT-MMP-2 staining was present in a filamentous granular pattern consistent with our previously reported mitochondrial localization [14]. Quantitative levels of MMP-2 isoform fluorescence intensity are shown in Fig 2, Panels III and IV, and confirm the impressions detailed above.

### The NF-κB inhibitor PDTC blocks NTT-MMP-2 transcription

**Fig 3:**
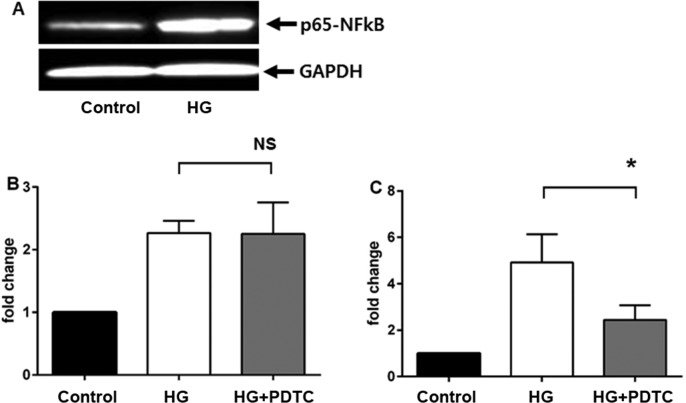
Effects of the NF-κB inhibitor, PDTC, on high glucose (30 mM) medium induction of FL-MMP-2 and NTT-MMP isoform expression. Panel A: Western blot of HK2 cell lysates at 48 hours of culture in control (5 mM) and high (30 mM) glucose concentrations. Compared to housekeeping GAPDH, there is a significant induction of NF-κB expression by culture in high glucose medium Panel B: Effect of the NF-κB inhibitor PDTC (50 μM) on FL-MMP-2 transcript levels measured by qPCR after 48 hours culture with control (5 mM) and high (30 mM) glucose. Treatment with PDTC does not significantly inhibit FL-MMP-2 expression (N = 3; p>0.05). Panel C: Effect of the NF-κB inhibitor PDTC (50 μM) on NTT-MMP-2 transcript levels measured by qPCR after 48 hours culture with control (5 mM) and high (30 mM) glucose. Treatment with PCTC significantly inhibits NTT-MMP-2 expression (N = 3; p<0.05).

First, we tested whether high glucose media upregulates NF-κB expression in HK2 cells. Western blot analysis showed that NF-κB expression increased greatly as compared with controls (Fig 3, Panel A). Inclusion of 50μM PDTC in the culture medium had no effect on high glucose-mediated expression of the FL-MMP-2 isoform, but did significantly suppress expression of the NTT-MMP-2 isoform to near basal levels (Fig 3, panels B, C). These observations suggest that high glucose-mediated induction of FL-MMP-2 and NTT-MMP-2 expression are mediated by distinct signaling pathways.

### The FL-MMP-2 and NTT-MMP-2 isoforms are induced in kidneys of diabetic mice

**Fig 4:**
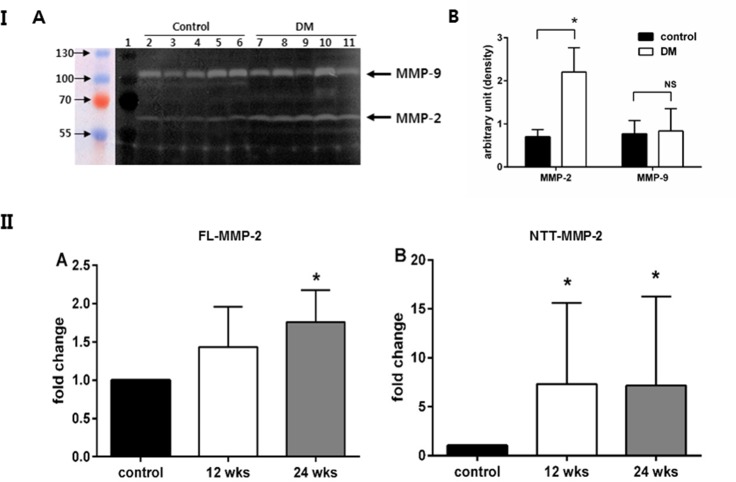
Induction of renal expression of FL-MMP-2 and NTT-MMP-2 in diabetic mice. **Diabetes mellitus was induced in C57/BL6 male mice by daily injection of streptozotocin (40 mg/kg) for five days.** Panel I A: Gelatin zymography of renal cortical extracts from control and diabetic mice (24 weeks). Zones of lysis denote gelatin enzymatic activity for MMP-9 (98 kDa) and MMP-2 (68 kDa). MMP-2 enzymatic activity is increased in the renal cortex of diabetic mice as compared to normoglycemic controls. There is no significant increase in MMP-9 activity in the renal cortex of diabetic mice as compared to normoglycemic controls. Panel I B: The band intensity was quantified by measuring pixel intensity. More MMP-2 enzymatic acitivities were noted in diabetic group as compared to control group. There is no increase in the lytic band corresponding to MMP-9 in the diabetic samples as compared to controls (*: p<0.05, NS: not significant). Panel II A: Quantitation of FL-MMP-2 transcript abundance in renal cortices of normoglycemic (control) and diabetic mice at 12 and 24 weeks. There is a modest, but significant increase in FL-MMP-2 transcript abundance after 24 weeks of diabetes (N = 8;* p<0.05). Panel II B: Quantitation of NTT-MMP-2 transcript abundance in renal cortices of normoglycemic (control) and diabetic mice at 12 and 24 week. NTT-MMP-2 transcript abundance is significantly increased at both 12 and 24 weeks of diabetes (N = 8; *p<0.05).

We generated diabetic mice using repetitive low dose streptozotocin injection. Murine kidneys were examined after 12 and 24 weeks of diabetes. Gelatin zymography demonstrated that total MMP-2 enzyme activity was increased in renal cortical extracts prepared from 24 week diabetic mice as compared to controls. Given the relatively limited resolution of gelatin zymography, with diffusion of areas of lysis, it is not possible to distinguish between the 68 kDa FL-MMP-2 isoform and the 65 kDa NTT-MMP-2 isoform using this technique (Fig 4, Panel I). In contrast, there was no change in the level of renal cortical expression of MMP-9. With quantitative PCR, FL-MMP-2 and NTT-MMP-2 transcripts were upregulated as compared with non-diabetic control mice. NTT-MMP-2 expression, in particular, was more strikingly increased as compared with FL-MMP-2 in 24 week diabetic mice (7.2±9.1 fold change, *p* = 0.003 vs. 1.8±0.4 fold change, *p* = 0.002, respectively) (Fig 4, Panel II).

### Immunohistochemical studies of murine diabetic kidneys

**Fig 5:**
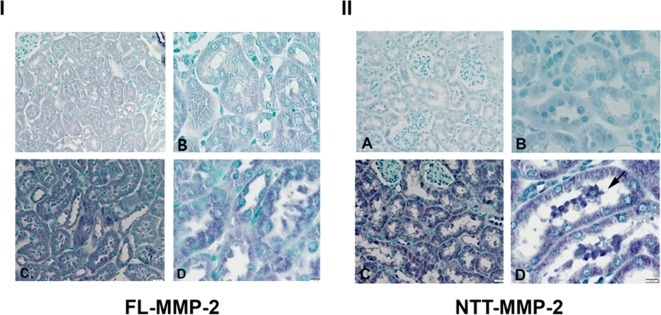
Immunohistochemical staining of renal cortices for FL-MMP-2 (panel I) and NTT-MMP-2 (panel II) of normoglycemic and diabetic mice (24 weeks). Panel I: There is detectable FL-MMP-2 in the renal cortex of control mice (A, B; x 200 and x 400 respectively) and this is increased in renal cortex of mice following 24 weeks of diabetes (C, D; x 200 and x 400 respectively). Panel II: NTT-MMP-2 protein expression is not detectable in the renal cortex of control mice (A, B; x 200 and x 400 respectively) but is greatly increased in the renal cortex of mice with 24 weeks of diabetes (C, D; x 200 and x 400 respectively). Note strong NTT-MMP-2 staining in necrotic tubular epithelial cells in tubular lumen of diabetic kidneys (D, arrow).

IHC staining for the FL-MMP-2 isoform revealed low, but detectable levels of protein expression in the proximal tubular epithelial cells of control mice (Fig 5I-A, B). FL-MMP-2 protein expression was increased in the tubular epithelial cells of diabetic mice (Fig 5I-C, D). NTT-MMP-2 immunostaining was not detected in the tubular epithelial cells of control mice (Fig 5II-A, B), but was markedly increased in the tubular epithelial cells of the diabetic mice, a finding consistent with the qPCR assessments (Fig 5II-C, 5D)

### Expression of MMP-2 isoforms in human diabetic kidney disease

**Fig 6:**
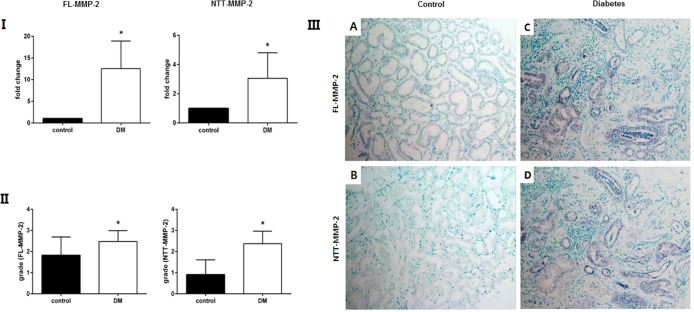
FL-MMP-2 and NTT-MMP-2 expression in renal biopsies of human controls and diabetic nephropathy. Panel I: qPCR measurement of FL-MMP-2 and NTT-MMP-2 transcript abundance in renal biopsies from controls and patients with diabetic nephropathy (DM). The transcript of both MMP-2 isoforms is significantly increased in the diabetic kidneys (N = 25; * p<0.05). Panel II: Semiquantitative scoring of immunohistochemistry staining of the FL-MMP-2 and NTT-MMP-2 isoforms as detailed in Materials and Methods. There is a statistically significant increase in both MMP-2 isoform protein expression in kidneys with diabetic nephropathy (N = 25; * p<0.05). Panel III: As compared to control kidney biopsies (A: FL-MMP-2; B: NTT-MMP-2) expression of both MMP-2 isoforms is increased in kidneys with diabetic nephropathy, particularly in dilated and atrophic tubules associated with interstitial inflammation (x 200).

In human diabetic kidney biopsies analyzed in this study proximal tubular injury, tubular dilatation, and tubular atrophy scoring were 2.4±0.7, 1.7±1.1, 2.1±0.9, respectively. Both isoforms of MMP-2 were highly expressed in human diabetic kidney tissue. Specifically, the FL-MMP-2 transcript abundance measured by qPCR was increased about by 12.5±6.4 times compared with normal control human kidney samples (Fig 6, Panel I). NTT-MMP-2 transcript was increased 3.1±1.7 fold (*p*<0.001). Immunohistochemical staining grade for both FL-MMP-2 and NTT-MMP-2 was higher in diabetic kidney disease as compared to controls (FL-MMP-2: 1.8±0.9 vs. 2.5±0.5, *p* = 0.03; NTT-MMP-2: 0.9±0.7 vs. 2.4±0.6, *p*<0.001) (Fig 6, Panel II). IHC staining for both the FL-MMP-2 and NTT-MMP-2 isoforms was most prominent within dilated tubules associated with tubulointerstitial mononuclear cell infiltration (Fig 6, panel III).

**Fig 7:**
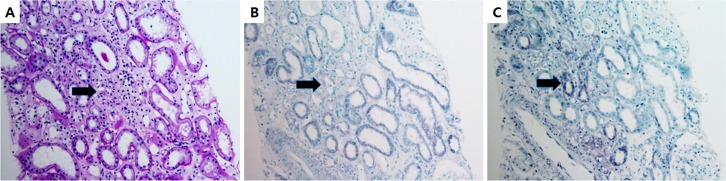
The FL-MMP-2 and NTT-MMP-2 isoforms are expressed within the same tubular segments. Serial sections of kidneys with diabetic nephropathy were stained with PAS (A) and by immunohistochemistry for FL-MMP-2 (B) and NTT-MMP-2 (C). Staining for both isoforms is prominent within dilated tubules and occurs in the same tubular segments (arrows, x200).

We performed IHC for the FL-MMP-2 and NTT-MMP-2 isoforms on serial sections of human diabetic renal tissue to determine whether these isoforms were expressed within the same tubular segments. Representative results are shown in Fig 7, and confirm co-expression of the two MMP-2 isoforms in the same tubular segments.

## Discussion

### Principal findings

The principal findings of the current study are the demonstration that two discrete MMP-2 isoforms are induced by a simulated diabetes mellitus milieu in vitro and in a widely used murine model of diabetic renal disease. Further, we confirmed expression of both the FL-MMP-2 and NTT-MMP-2 isoforms in archival renal biopsy specimens of human diabetic nephropathy. Both isoforms are expressed within the same tubular segments and these frequently demonstrated evidence for tubular injury, including dilatation and inflammation.

### The specific role(s) of matrix metalloproteinases in diabetic nephropathy are undefined

We note that there is a large and very inconsistent literature concerning the expression of multiple individual matrix metalloproteinases in both experimental and clinical diabetic nephropathy [15]. This inconsistency also applies to the specific matrix metalloproteinase, MMP-2. Potential explanations derive from cell culture experiments employing a wide variety of conditions and cell types, as well as from the use of widely varying animal models of diabetes mellitus. Further, much of the attention, at least in terms of human diabetic nephropathy, has been placed in changes in matrix metalloprotease expression in glomeruli rather than on tubular epithelial cell injury and inflammation [15].

### Characterization of the NTT-MMP-2 isoform

We initially characterized the NTT-MMP-2 isoform in isolated mitochondria from a murine model of systolic heart failure and accelerated atherogenesis [11]. In addition, we detected the NTT-MMP-2 isoform in mitochondria from aged mice. We demonstrated that the NTT-MMP-2 isoform is generated by oxidative stress-mediated activation of an alternate promoter located within the first intron of the MMP-2 gene and that translation is initiated from M^77^ in the second exon. The NTT-MMP-2 isoform is present within the mitochondrial intramembranous space and induces mitochondrial-nuclear stress signaling with induction of a defined transcriptome of pro-inflammatory innate immunity and cell death-associated genes [11].

### Relationship between MMP-2 expression and oxidative stress

We recently reported that both MMP-2 isoforms are expressed in archival biopsies from patients with renal transplant delayed graft function, and that expression of the NTT-MMP-2 isoform was most closely linked with tubular epithelial cell necrosis [14]. Oxidative stress is a common feature of renal transplant delayed graft function, which represents a defined form of acute ischemia/reperfusion injury. Prior transcriptional regulatory studies of the FL-MMP-2 promoter have shown that transcription is potently enhanced in the setting of oxidative stress by an AP-1 binding enhancer [16–18]. Similarly, the NTT-MMP-2 alternate promoter is activated primarily by oxidative stress via an NF-κB enhancer element (DHL, unpublished observations), which presumably explains the selective suppression of NTT-MMP-2 transcription by the anti-oxidant, NF-κB inhibitor, PDTC.

Within the setting of experimental and human diabetes mellitus, acute and chronic hyperglycemia are associated with enhanced oxidative stress in multiple organs, including the kidney [12, 13]. Tubular epithelial cell injury, with resultant tubular atrophy, peritubular inflammation and fibrosis is a common feature of diabetic nephropathy and is associated with an enhanced sensitivity to superimposed ischemia/reperfusion injury [19]. We noted that tubular epithelial cell expression of the MMP-2 isoforms occurred in the same renal tubular segments which frequently showed evidence for tubule cellular injury and inflammation. Based on our prior studies with renal tubule-specific transgenic expression of the FL-MMP-2 isoform, we suggest that the final pathologic phenotype of diabetic nephropathy is induced by the combined actions of the FL-MMP-2 isoform on the tubular basement membrane and on the induction of tubular cell necrosis and inflammation by the NTT-MMP-2 isoform.

### Potential therapeutic aspects of the current study

Our work suggests that selective targeting of the two MMP-2 isoforms could represent a potential novel therapeutic approach for the prevention or treatment of diabetic nephropathy, in addition to the recognized approaches of stringent control of blood glucose and lipids. While clinical trials primarily using anti-oxidant vitamins have not shown benefit, it is possible that anti-oxidant strategies employing more potent agents, such as PDTC, could possibly have benefit through the inhibition of NTT-MMP-2 synthesis.

### Limitations

The current study is based on an analysis of FL-MMP-2 and NTT-MMP-2 expression in an in vitro model of diabetes mellitus using HK-2 tubular epithelial cells and with the streptozotocin murine model of Type I diabetes mellitus. Further, our human renal biopsy archival studies were not powered to determine a relationship between MMP-2 isoform expression and clinical outcomes. Future studies are planned to address these important issues and to validate the MMP-2 isoforms as high yield therapeutic targets.
